# Orphan Drug Use in Patients With Rare Diseases: A Population-Based Cohort Study

**DOI:** 10.3389/fphar.2022.869842

**Published:** 2022-05-16

**Authors:** Francesca Gorini, Michele Santoro, Anna Pierini, Lorena Mezzasalma, Silvia Baldacci, Elena Bargagli, Alessandra Boncristiano, Maurizia Rossana Brunetto, Paolo Cameli, Francesco Cappelli, Giancarlo Castaman, Barbara Coco, Maria Alice Donati, Renzo Guerrini, Silvia Linari, Vittoria Murro, Iacopo Olivotto, Paola Parronchi, Francesca Pochiero, Oliviero Rossi, Barbara Scappini, Andrea Sodi, Alessandro Maria Vannucchi, Alessio Coi

**Affiliations:** ^1^ Unit of Epidemiology of Rare Diseases and Congenital Anomalies, Institute of Clinical Physiology, National Research Council, Pisa, Italy; ^2^ Respiratory Diseases Unit, Department of Medical and Surgical Sciences and Neurosciences, University of Siena, Siena, Italy; ^3^ Neuroscience Department, A. Meyer Children Hospital-University of Florence, Florence, Italy; ^4^ Department of Clinical and Experimental Medicine, University of Pisa, Pisa, Italy; ^5^ Cardiomyopathy Unit, Careggi University Hospital, University of Florence, Florence, Italy; ^6^ Center for Bleeding Disorders and Coagulation, Department of Oncology, Careggi University Hospital, Florence, Italy; ^7^ Hepatology Unit, University Hospital of Pisa, Pisa, Italy; ^8^ Metabolic and Muscular Unit, A. Meyer Children Hospital, Florence, Italy; ^9^ Department of Neuroscience, Psychology, Drug Research and Child Health, University of Florence, Careggi University Hospital, Florence, Italy; ^10^ Department of Experimental and Clinical Medicine, SOD Immunologia e Terapie Cellulari, Careggi University Hospital, University of Florence, Florence, Italy; ^11^ Immunuallergology Unit, SOD Immunoallergologia, Careggi University Hospital, Florence, Italy; ^12^ Hematology Unit, Careggi University Hospital, Florence, Italy; ^13^ Center Research and Innovation of Myeloproliferative Neoplasms (CRIMM), Department of Experimental and Clinical Medicine, Careggi University Hospital, University of Florence, Florence, Italy

**Keywords:** orphan drug, rare disease, registry, population-based, prescription

## Abstract

**Background:** Orphan drugs are used for the diagnosis, prevention and treatment of rare diseases that, in the European Union, are defined as disorders affecting no more than 5 persons in 10,000. So far, a total of around 800 orphan medicinal products have been approved by the European Medicines Agency, however the utilization profile of orphan drugs has yet to be explored. This study aimed at assessing the utilization profile of orphan drugs authorized for marketing by the Italian Medicines Agency using population-based data.

**Methods:** A total of 21 orphan drugs used in outpatient settings, approved in the European Union before or during the 2008–2018 period and involving 15 rare diseases, were included in the study. The monitored population included patients with one of the conditions surveilled by the population-based Tuscany Registry of Rare Diseases and diagnosed between 2000–2018. A multi-database approach was applied, by linking data from the registry with information collected in drug prescriptions databases. The prevalence and intensity of use were estimated for the selected orphan drugs and other non-orphan medications, used to treat the same rare disease and for which a change in the prevalence of use was hypothesized after authorization of the orphan drug.

**Results:** For some diseases (acquired aplastic anemia, tuberous sclerosis complex, most metabolic diseases) a low prevalence of orphan drugs use was observed (range between 1.1–12.5%). Conversely, orphan drugs were frequently used in hemophilia B, Wilson disease and idiopathic pulmonary fibrosis (maximum of 78.3, 47.6 and 41.8%, respectively). For hemophilia B and Leber’s hereditary optic neuropathy, there are currently no other medications used in clinical practice in addition to orphan drugs. Six orphan drugs were used for the treatment of pulmonary arterial hypertension, appearing the elective therapy for this disease, albeit with different utilization profiles (range of prevalence 1.7–55.6%).

**Conclusion:** To the best of our knowledge, this is the first study investigating the utilization profile of orphan drugs prescribed in a defined geographical area, and providing relevant information to monitor over time potential changes in the prevalence of these medications as well as in the health care decision making.

## Introduction

Orphan drugs are used for the diagnosis, prevention and treatment of rare diseases. According to the European Union (EU) Regulation on orphan medicinal products (1999), a rare disease is one that affects no more than five persons in 10,000 (European Union, 1999). To date, between 6,000 and 8,000 different rare diseases have been identified (European Commission, 2020), most of which include chronically debilitating conditions that can lead to premature death (Nguengang Wakap et al., 2020). Rare diseases are individually rare but their global point prevalence is estimated to account for 3.5–5.9% (Nguengang Wakap et al., 2020), which equates to 263–446 million persons worldwide and 27–36 million people in the EU (Davies et al., 2017; Shourick et al., 2021).

Following the Orphan Drug Act approval in the United States (US) (Department of Health and Human Services, 1983), the EU established specific legislations to encourage research on orphan drugs for specific rare diseases (Melnikova, 2012; Franco, 2013). In particular, a total of around 800 orphan medicinal products have been approved by the European Medicines Agency (EMA) and the United States Food and Drug Administration for the treatment of rare diseases (EMA, 2020; National Organizations for Rare Disorders, 2021).

As for the EU, although orphan drug market authorization is a centralized procedure, the health technology assessment, pricing, and reimbursement are governed at national level (Czech et al., 2020). In Italy, the national healthcare system currently reimburses almost 80 orphan drugs (Italian Medicines Agency, 2022) although, when the EMA’s marketing authorization is not available, a patient suffering from a rare disease can still access a medication through the compassionate use program (Law no. 326/2003) and other national acts (Law no. 648/1996 and Law no. 94/1998) (Italian Medicines Agency, 2021).

To date, the utilization profile of orphan drugs has not been addressed by specific studies, except for the analysis of Stolk et al. (2009) who compared the use of five orphan drugs across different European countries using Defined Daily Doses (DDDs) per 1,000 persons per year.

In this study, we evaluated the use of orphan drugs authorized for marketing by the Italian Medicines Agency using population-based data. We applied a multi-database approach, by integrating data from the population-based Tuscany Registry of Rare Diseases in Italy with data collected in the regional drug prescription databases.

## Methods

### Study Design, Data Source and Study Subjects

This retrospective cohort study covered the period 2008–2018. The monitored population included patients residing in Tuscany, an Italian region of 3,701,343 inhabitants (source: Italian National Institute of Statistics as of 1 January 2018) and diagnosed between 1 January 2000 and 31 December 2018 with one of the rare diseases surveilled by the population-based Tuscany Registry of Rare Diseases according to the Italian Law (Decree of the President of the Council of Ministers, 01/2017), and for which there is an exemption from co-payment. The registry is based on a regional network allowing the detection of all cases diagnosed at any age by any of the regional health centres, and is one of the main contributors to the National Centre of Rare Diseases of the Italian National Institute of Health (Coi et al., 2017).

Prescriptions were retrieved by Tuscany drug database that collects the prescriptions of drugs dispensed by community and hospital pharmacies for outpatient use. Orphan drugs authorized for marketing by the Italian Medicines Agency before 2008 or during the study period, were selected for this analysis (Italian Medicines Agency, 2018). Orphan drugs for which there were only prescriptions in the last year of the study period or which were authorized at the end of the study period were not included in the study.

Rare diseases for which the selected orphan drugs have therapeutic indications were included in the study; diseases with less than 5 total patients diagnosed, were excluded. Hence, the study was carried out on a total of 21 orphan drugs involving 15 rare diseases, for a total of six different groups of disease (Table 1). Eight of the 21 orphan drugs concluded the 10-year period of market exclusivity (an incentive awarded by the European Commission to protect authorized orphan drugs from competition from similar medicines with similar indications; EMA, 2022) during the study period as follows: deferasirox, eltrombopag, miglustat, zinc acetate, sildenafil, iloprost, bosentan and ambrisentan.

**TABLE 1:** List of rare diseases and orphan drugs included in the study.

Rare disease by nosological group	Total cases 2000–2018	Orphan drug (ATC code)	Issue date of marketing authorization by the Italian Medicines Agency
Diseases of the blood and hematopoietic organs			
Hemophilia B	32	Coagulation factor IX (B02BD04)	27/08/1997 (Nonacog alfa)
			11/05/2016 (Albutrepenonacog alfa)
			12/05/2016 (Eftrenonacog alfa)
Β-thalassemia	115	Deferasirox (V03AC03)	28/08/2006
Acquired aplastic anemia	20	Eltrombopag (B02BX05)	11/03/2010
Metabolic diseases			
Urea cycle disorders	27	Sodium phenylbutyrate (A16AX03)	08/12/1999
Methylmalonic acidemia	36	Carglumic acid (A16AA05)	01/06/2011
Phenylketonuria	116	Sapropterin dihydrochloride (A16AX07)	02/12/2008
Gaucher disease type I	16	Eliglustat (A16AX10)	19/01/2015
		Miglustat (A16AX06)	20/11/2002
Fabry disease	89	Migalastat (A16AX14)	26/05/2016
Wilson disease	24	Zinc acetate (A16AX05)	13/10/2004
Leber’s hereditary optic neuropathy	53	Idebenone (N06BX13)	08/09/2015
Immune system disorders			
Hereditary angioedema	43	Icatibant (B06AC02)	11/07/2008
Respiratory diseases			
Idiopathic pulmonary fibrosis	644	Pirfenidone (L04AX05)	28/02/2011
Pulmonary arterial hypertension	114	Sildenafil (G04BE03)	28/10/2005
		Iloprost (B01AC11)	16/09/2003
		Bosentan (C02KX01)	14/05/2002
		Ambrisentan (C02KX02)	21/04/2008
		Macitentan (C02KX04)	20/12/2013
		Riociguat (C02KX05)	27/03/2014
Peripheral and central nervous system diseases			
Lennox-Gastaut syndrome	22	Rufinamide (N03AF03)	16/01/2007
Congenital anomalies, chromosomal aberrations and genetic syndromes			
Tuberous sclerosis complex	75	Everolimus (L01XE10)	02/09/2011

### Study Outcomes and Data Analysis

The Anatomical Therapeutic Chemical (ATC) classification system was used to code drug information. The prevalence and intensity of use were estimated for the selected orphan drugs. Furthermore, non-orphan medications, used to treat the same rare disease and for which a change in the prevalence of use was hypothesized after the orphan drug was authorized, were also evaluated for comparison.

For each disease, prevalent cases at 1st January were calculated per each year of the study period through the linkage of the cases of the Registry to the regional vital statistics containing civil registrations data. All patients with rare diseases endowed by a unique regional identification code were anonymously linked to the regional drug prescriptions databases.

The prevalence of use is a measure of exposure and was calculated by year in the study period, as the ratio between the number of cases with at least one prescription and the number of prevalent cases at the beginning of each year (Carrie et al., 2006; Coi et al., 2021). In this study, the intensity of use, a measure of drug burden, was calculated by dividing the total number of prescriptions of each medication for the total number of users (cases with at least one dispensing per year) (Da Cas et al., 2020; Italian Medicines Agency, 2020). The prescriptions per user (Pr/Us) indicator is less subject to bias compared to the DDDs per user which, being based on the average number of days of therapy, it can be influenced by extreme values of the DDD distribution and, furthermore, using this latter indicator a bias may also occur for drug classes with chronic use.

## Results

The results of the study are presented hereinafter based on the classification of rare diseases by nosological group.

### Diseases of the Blood and Hematopoietic Organs

**FIGURE 1:**
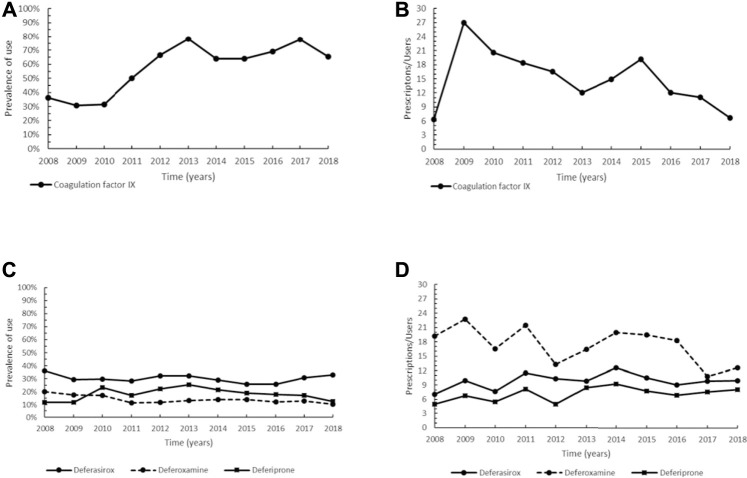
Trend of prevalence (graphs **(A,C)**) and intensity of use (graphs **(B,D)**) of the coagulation factor IX indicated for hemophilia B and of the orphan drug deferasirox and deferoxamine and deferiprone indicated for β-thalassemia Prevalence of use: ratio between the number of cases with at least one prescription and the number of prevalent cases at the beginning of each year. Prescription/users: ratio between the number of prescriptions of each medication for the total number of patients with at least one dispensing per year.

The orphan drugs nonacog alfa, eftrenonacog alfa and albutrepenonacog alfa (ATC: B02BD04) and the non-orphan medication nonacog gamma (ATC: B02BD04) are recombinant concentrates that replace the missing coagulation factor IX (FIX) in hemophilia B. An increasing prevalence trend was observed between 2011 and 2013, achieving a maximum of use in 2013, with values varying between 50.0% and 78.3% for most of the study period (Figure 1A). The intensity of use, after reaching a peak in 2009 (27.0 Pr/Us), showed a progressive decline (Figure 1B).

As regards β-thalassemia major and intermedia, the oral chelators deferasirox (ATC: V03AC03), a drug with the orphan designation, deferiprone (ATC: V03AC02) and deferoxamine (ATC: V03AC01) were here considered. Deferasirox had the highest prevalence of use, showing a steady trend (approximately 30%) over the study period (Figure 1C). The trend of deferoxamine showed a maximum in 2008 (20.0%), then steady values since 2011, while deferiprone, after reaching a peak in 2013 (25.3%), progressively declined until 2018. Figure 1D illustrates the intensity of use of deferasirox and the two non-orphan medications. The former displayed a slightly growing trend since 2011, with values between 9.0 and 12.6 Pr/Us. A similar profile, but with lower values, was observed for deferiprone. Conversely, an overall greater intensity of use was found for deferoxamine, although with a decreasing trend since 2015.

Eltrombopag (ATC: B02BX05), an orphan drug authorized for marketing by EMA in 2010, is an oral thrombopoietin receptor agonist indicated for adult patients with acquired aplastic anemia (SAA). Besides, cyclosporin A (ATC: L04AD01), an immunosuppressive agent with the same therapeutic indications, was also here evaluated as a comparison with eltrombopag. Horse antithymocyte globulin, another immunosuppressant, was not included in the analysis being a medication administered in inpatient settings, therefore not traceable by our databases (see *Methods* for inclusion/exclusion criteria). In Tuscany, eltrombopag has been used since 2016, showing a prevalence ranging from 8.3% to 12.5% (data not plotted due to few years of estimated prevalence). An elevated prevalence of use for cyclosporine A was seen in 2010–2015 (range 50.0–100.0%) but, concurrently with the introduction of eltrombopag, a decreasing trend has been observed from 2016 onwards. The intensity of use of eltrombopag ranged between 3.0 and 11.0 Pr/Us, while cyclosporine A overall showed a high variability with values between 10.3–12.7 Pr/Us in the same period.

### Metabolic Disorders

**FIGURE 2:**
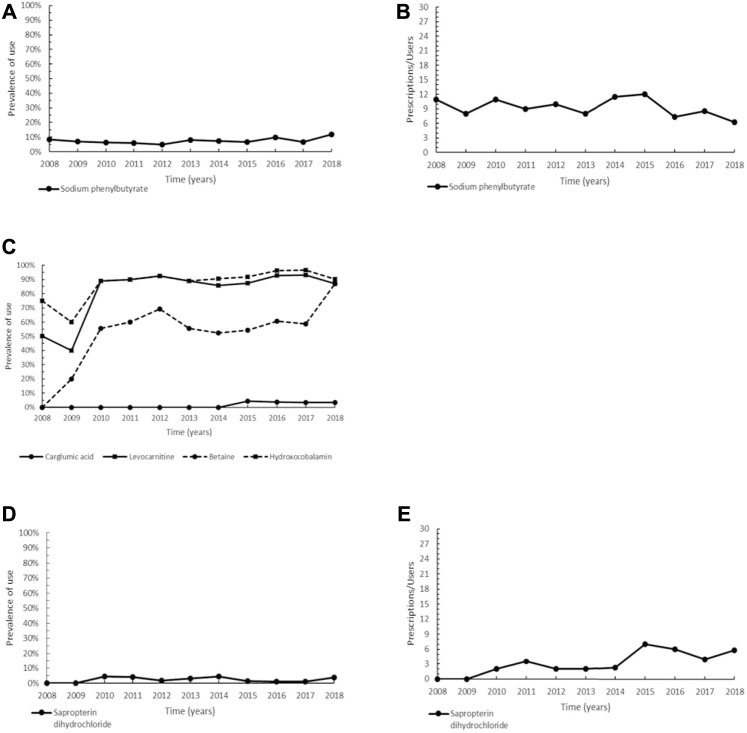
Trend of prevalence (graphs **(A,C,D)**) and intensity of use (graphs **(B,E)**) of sodium phenylbutyrate indicated for urea cycle disorders, of the orphan drug carglumic acid and levocarnitine, betaine and hydroxycobalamin indicated for methylmalonic acidemia, and of sapropterin dihydrochloride indicated for phenylketonuria. Prevalence of use: ratio between the number of cases with at least one prescription and the number of prevalent cases at the beginning of each year. Prescription/users: ratio between the number of prescriptions of each medication for the total number of patients with at least one dispensing per year.

The orphan drugs that were granted market authorization in the EU for long-term treatment of urea cycle disorders (UCDs) under the period of investigation are sodium phenylbutyrate (NaPB; ATC: A16AX03) and glycerol phenylbutyrate (ATC: A16AX09), but for the latter, approved in 2015, there were no prescriptions in our drug databases during the study period. In our study, the prevalence of use of NaPB remained at low values (5.0–11.8%) for the whole period (Figure 2A). The intensity of use of NaPB showed a slight decreasing trend with values from 12.0 Pr/Us in 2015 to 6.3 Pr/Us in 2018 (Figure 2B). Unfortunately, it was not possible to evaluate the use of the other previously approved non-orphan medicinal products for UCDs (sodium benzoate and sodium phenylacetate), because of their intravenous administration in inpatient settings.

Carglumic acid (N-carbamylglutamate, ATC: A16AA05) is an oral orphan drug authorized from the EU for the treatment of hyperammonaemia caused by methylmalonic acidemia (MMA). Other non-orphan medications normally used in both acute and chronic management of MMA were here compared to carglumic acid: levocarnitine (ATC: A16AA01), hydroxocobalamin (ATC: A16AA03), and betaine (ATC: B03BA06). Authorized in 2011, carglumic acid has exclusively been administered in inpatients for many years, therefore we only observed prescriptions of carglumic acid from 2015 onwards, with a steady trend and low prevalence rates (range 3.2–4.2%). Levocarnitine and hydroxocobalamin showed a constantly high prevalence of use (range between 87.1–96.6% in the years 2015–2018), while the trend of betaine was characterized by lower values (between 54.2 and 87.1% in the same period) (Figure 2C). Overall, these drugs have different therapeutic indications based on clinical signs and symptoms, the underlying enzymatic defect, the acute or chronic presentation of MMA, thus an assessment of their intensity of use was not carried out.

The two orphan drugs approved by the EMA for the treatment of phenylketonuria (PKU) are sapropterin dihydrochloride (ATC: A16AX07) and pegvaliase (ATC: A16AB19); the latter, however, could not be assessed as authorized for marketing in 2019. In our study, we observed a steady trend for sapropterin dihydrochloride from 2010 (first year of use) to 2018 (range 1.1–4.5%) (Figure 2D). The profile of intensity of use was characterized by steady values from 2010 to 2014, then increased from 2015 onwards (Figure 2E).

For Gaucher disease type I (GDI), which represents 99% of GD patients with Gaucher disease in our cohort, two therapeutic approaches are currently available: enzyme replacement therapy (ERT) and substrate reduction therapy (SRT). Miglustat (ATC: A16AX06) and eliglustat (ATC: A16AX10) are the two orphan drugs representing the SRT and approved by the EU for the treatment of GDI. In our patient population, miglustat had prescriptions in the years 2009–2010 with a prevalence of use in the range 10.0–12.5%, and then it was no longer used, whereas prescriptions for eliglustat were only registered in 2018, with a prevalence of use of 7.7% (data not plotted, only two and 1 year of estimated prevalence, respectively). The intensity of use recorded for miglustat varied between 2.0 and 7.0 Pr/Us, while for eliglustat was 2.0 Pr/Us. ERT medications were not analyzed in the present study because of their main administration in inpatients (see *Methods* for inclusion/exclusion criteria).

Migalastat (ATC: A16AX14) is the only orphan drug orally administered for the treatment of Fabry disease and authorized for marketing in the EU. In this study, migalastat was prescribed from 2017 onwards, with a maximum of prevalence of 11.9% in 2018 (data not plotted, only 2 years of estimated prevalence). As regards the intensity of use, 7.1 Pr/Us were observed in the last year of the study period.

**FIGURE 3:**
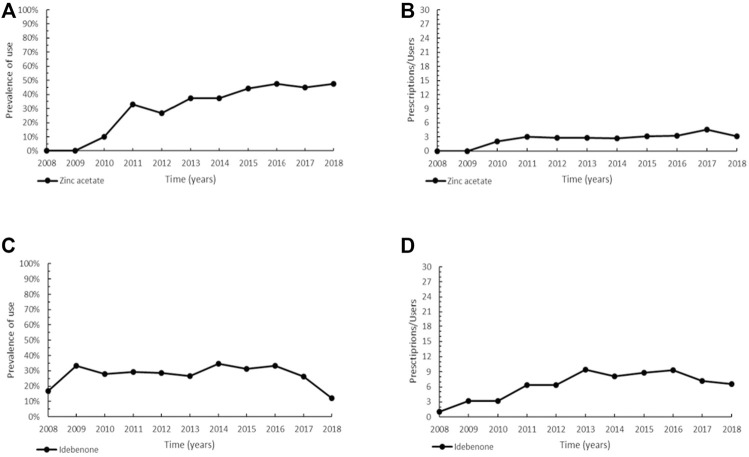
Trend of prevalence (graphs **(A,C)**) and intensity of use (graphs **(B,D)**) of zinc acetate indicated for Wilson disease and of idebenone indicated for Leber’s hereditary optic neuropathy. Prevalence of use: ratio between the number of cases with at least one prescription and the number of prevalent cases at the beginning of each year. Prescription/users: ratio between the number of prescriptions of each medication for the total number of patients with at least one dispensing per year.

Zinc acetate (ATC: A16AX05) is a medication that was granted an orphan designation and used as a treatment option in Wilson disease (WD). In this study, the prevalence of zinc acetate showed an overall increasing trend over the entire period up to reach 47.6% in 2018 (Figure 3A). As for the intensity of use, the trend was characterized by generally steady values of Pr/Us (Figure 3B). Other drugs able of reducing copper overload in the body by increasing urinary copper excretion are the chelators D-penicillamine and trientine dihydrochloride. Unfortunately, it has not been possible to assess the prevalence of use of the non-orphan medicine D-penicillamine, because since 2011 it has been directly produced and distributed by the Italian military chemical pharmaceutical plants on the basis of specialist prescriptions that are not collected in our databases. Likewise, trientine dihydrochloride, indicated for the treatment of Wilson’s disease in patients intolerant to D-penicillamine therapy, has never been prescribed in our patient population.

Idebenone (ATC: N06BX13), designated as orphan in 2007 and authorized to marketing in the EU since 2015, represents the current and only medication approved for the treatment of Leber’s hereditary optic neuropathy (LHON) in adults and adolescents aged 12 years and over. Patients of our cohort have been prescribed idebenone since 2008, before marketing authorization, through the compassionate use program. The prevalence trend showed an increase in 2009, to maintain almost steady values (maximum of 34.5% in 2014), followed by a decline in the years 2017–2018 (Figure 3C). As depicted in Figure 3D, the profile of intensity of use increased since 2011, followed by a steady trend.

### Immune System Disorders

**FIGURE 4:**
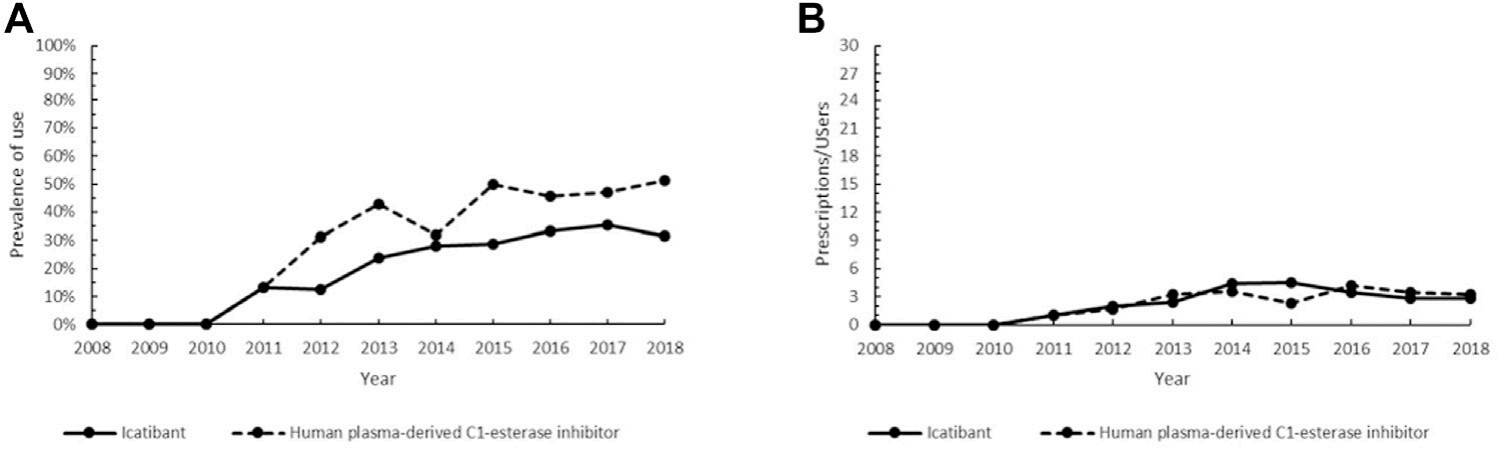
Trend of the prevalence (graph **(A)**) and intensity of use (graph **(B)**) of the orphan drug icatibant and the human plasma-derived C1-esterase inhibitor indicated for hereditary angioedema. Prevalence of use: ratio between the number of cases with at least one prescription and the number of prevalent cases at the beginning of each year. Prescription/users: ratio between the number of prescriptions of each medication for the total number of patients with at least one dispensing per year.

Icatibant (ATC: B06AC02), an orphan drug introduced in Italy in 2008, is a synthetic peptide, which belongs to on-demand therapies for the treatment of acute attacks of hereditary angioedema (HAE)in adults, adolescents and children aged over 2 years. In our study, icatibant showed an increasing trend from 2011 to 2014 followed by an almost steady prevalence (range 28.6–35.3%) until the end of the study period (Figure 4A). The intensity of use of icatibant reached a maximum in 2015 (4.5 Pr/Us) and then slightly decreased up to 2.8 Pr/Us (Figure 4B). Using plasma-derived C1-esterase inhibitor (pdC1-INH; ATC: B06AC01), an on-demand conventional drug, as a comparison, we found that the prevalence of pdC1-INH increased more markedly reaching its peak in 2018 (51.4%) (Figure 4A), while its intensity of use was like that of icatibant (Figure 4B).

### Respiratory Diseases

**FIGURE 5:**
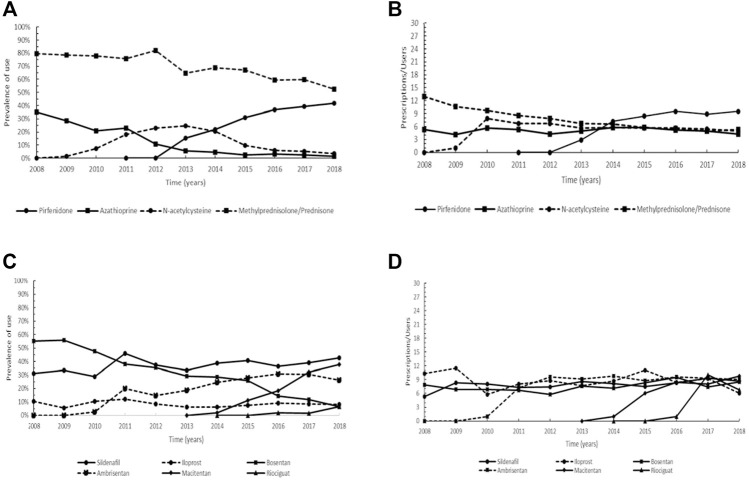
Trend of the prevalence (graphs **(A,C)**) and intensity of use (graphs **(B,D)**) of the orphan drug pirfenidone and azathioprine, methylprednisolone/prednisone, and N-acetylcysteine indicated for idiopathic pulmonary fibrosis and of bosentan, ambrisentan, macitentan, sildenafil, riociguat, and iloprost indicated for pulmonary arterial hypertension. Prevalence of use: ratio between the number of cases with at least one prescription and the number of prevalent cases at the beginning of each year. Prescription/users: ratio between the number of prescriptions of each medication for the total number of patients with at least one dispensing per year.

The orphan drugs approved for the treatment of idiopathic pulmonary fibrosis (IPF), are pirfenidone (ATC: L04AX05) and nintedanib (ATC: L01EX09). In our patient cohort, the use of pirfenidone, authorized in the EU in 2011, showed a continuous increasing trend from 2014 to 2018, with the higher percentage of users in the last year (prevalence of 41.8%) (Figure 5A). The intensity of use of pirfenidone increased between 2014 and 2016, followed by a steady trend (Figure 5B). Unfortunately, it was not possible to analyze the utilization profile of nintedanib, as this drug was not yet commercially available in Italy in the study period. In comparison with pirfenidone, we also investigated the trend of use of three non-orphan conventional medications: the immunosuppressant azathioprine (ATC: L04AX01), the glucocorticoids methylprednisolone (ATC: H02AB04) and prednisone (ATC: H02AB07), and the mucolytic N-acetylcysteine (ATC: R05CB01). In our cohort, azathioprine showed a decreasing trend, ranging from a maximum of 35.2% in 2008 to a minimum of 1.4% in 2018. After reaching a maximum in 2013 (24.8%), the prevalence of N-acetylcysteine also decreased, while the glucocorticoids methylprednisolone and prednisone were characterized by a steady trend in the years 2008–2012 (range 75.9–81.9%), followed by a decrease until 2018 (Figure 5A). As regards the intensity of use, azathioprine showed approximately steady values (4.1–5.8 Pr/Us), while for N-acetylcysteine a slight decreasing trend was observed from 2012 to 2018. The intensity of use of glucocorticoids varied from a maximum of 13.0 Pr/Us in 2008 to a minimum of 5.3 Pr/Us in 2017 (Figure 5B).

Overall, six drugs that obtained an orphan designation are employed for the treatment of patients with pulmonary arterial hypertension (PAH): bosentan (ATC: C02KX01), ambrisentan (ATC: C02KX02), macitentan (ATC: C02KX04), sildenafil (ATC: G04BE03), riociguat (ATC: C02KX05) and iloprost (ATC: B01AC11). In our cohort, bosentan was the most used in the early study period (prevalence of 55.6% in 2009) but showed a substantial decreasing trend from 2010 to 2018. Ambrisentan and macitentan displayed no prescriptions before 2010 and 2014, respectively and while ambrisentan was characterized by an increasing trend up to 2016 (reaching a prevalence of use of 30.9%), for macitentan a continuous increasing trend was observed until the end of the study period (maximum of 38.7% in 2018). Regarding sildenafil, a steady trend was observed with a maximum of 46.0% achieved in 2011, and a similar trend was found for iloprost, albeit with a lower prevalence (maximum of 12.0% in 2011). Riociguat had prescriptions only in the years 2016–2018, having been authorized to marketing in Italy in 2014 (Figure 5C). The graph on intensity of use showed that the trend of bosentan remained nearly steady throughout the study period and similarly, ambrisentan, after an increase in the years 2011–2012, ranged between 8.8 and 9.6 Pr/Us. For macitentan steady values were observed from 2016 onwards (9.8 Pr/Us in 2018), and generally steady values were also detected for sildenafil (maximum of 9.3 Pr/Us reached in 2017). A similar trend was shown by iloprost, while riociguat reached a maximum of 10.0 Pr/Us in 2017 (Figure 5D).

### Peripheral and Central Nervous System Diseases

**FIGURE 6:**
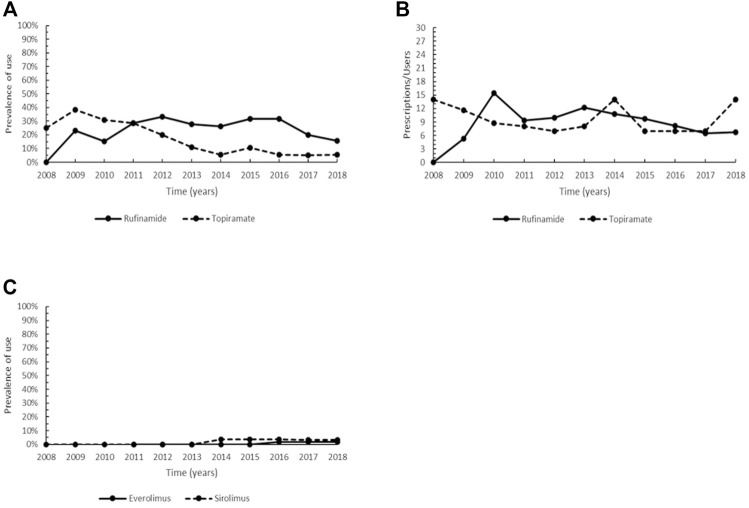
Trend of the prevalence (graph **(A)**) and intensity of use (graph **(B)**) of the orphan drug rufinamide and topiramate indicated for Lennox-Gastaut syndrome and trend of the prevalence (graph **(C)**) of the orphan drug everolimus and sirolimus indicated for tuberous sclerosis complex. Prevalence of use: ratio between the number of cases with at least one prescription and the number of prevalent cases at the beginning of each year. Prescription/users: ratio between the number of prescriptions of each medication for the total number of patients with at least one dispensing per year.

Rufinamide (ATC: N03AF03) was designated as orphan drug in 2004 for Lennox-Gastaut syndrome and authorized to marketing in the EU in 2007. In our cohort, rufinamide was prescribed from 2009 onwards, with an increase in the prevalence of use since 2011, followed by a steady prevalence until 2016 (range 27.8–31.6%) and a decrease in the last 2 years of the study period, when the same levels of 2009–2010 were observed (Figure 6A). The non-orphan drug topiramate (ATC: N03AX11), was also studied for comparison with rufinamide. Topiramate showed a decreasing trend from 2010 to 2014, subsequently settling on steady values (range 5.0–5.3%) (Figure 6A). The trend of intensity of use of the two medications was very similar, with values between 5.3 and 15.5 Pr/Us per year (Figure 6B).

### Congenital Anomalies, Chromosomal Aberrations and Genetic Syndromes

Currently approved medications for the treatment of tuberous sclerosis complex (TSC) are the orphan drug everolimus (ATC: L01XE10) and the non-orphan medicinal product sirolimus (ATC: L04AA10).

Everolimus was authorized for marketing in the EU in 2011 and there were no prescriptions of this drug in our cohort until 2014. Steady use was observed in the years 2016–2018, albeit with a very low prevalence (1.7–1.8% of patients with at least one prescription per year). A low and similar pattern was also observed for sirolimus, which showed a prevalence of use ranging between 3.4–3.8% in the years 2014–2018 (Figure 6C). Everolimus achieved the maximum intensity of use in 2017–2018 (6.0 Pr/Us), while a large variability was observed for sirolimus (range 1.5–15.5 Pr/Us) (data not plotted due to the low number of users).

## Discussion

### Diseases of the Blood and Hematopoietic Organs

Recombinant FIX concentrates are currently used to prevent and treat bleeding in patients with hemophilia B, an X-linked bleeding disorder caused by FIX deficiency (Rocca et al., 2011; EMA et al., 2021a, 2021b, 2021c, 2021d). In the past, other non-orphan FIXs were also used, i.e., plasma-derived products and, since 2016 long-acting coagulation factors became available, with the advantage of reducing frequency of injections and improving adherence to individualized treatment for hemophilia B (Shapiro, 2013). The observed reduction in the intensity of use is probably because of the increasing use of long-acting coagulation factors eftrenonacog alfa and albutrepenonacog alfa replacing recombinant nonacog alfa and gamma concentrates with shorter half-life (Ar et al., 2019; Srivastava et al., 2020) (Figure 1B). The introduction of long-acting coagulation factors has decreased the number of infusions for prophylaxis by 50–70%, with improved quality of life for the patients (Castaman, 2018). All currently available recombinant FIX concentrates have demonstrated similar efficacy in the treatment and prophylaxis of bleeding episodes and have a better safety profile compared to plasma-derived products that theoretically are not completely free in terms of viral and prion transmission (Alamelu et al., 2014; Rocino et al., 2014; Porteus, 2017; Castaman, 2018).

In patients with β-thalassemia major and intermedia, which originate from mutations on both genes encoding β globin chains, albeit with different clinical manifestations, deferasirox is the orphan drug employed in the prevention of iron overload-induced complications resulting from blood transfusions, necessary for the treatment of these diseases (Al-Kuraishy et al., 2017; Manglani et al., 2017). Deferiprone was the first iron oral chelator to be used, while deferoxamine has been considered the standard treatment for iron overload during the four past decades (Xia et al., 2013; Bollig et al., 2017). Deferasirox does not show more efficacy than deferoxamine, however it may represent a preferable treatment choice for patients manifesting intolerance or poor adherence to deferoxamine (Xia et al., 2013; Bollig et al., 2017). This might explain the overall higher prevalence of use of deferasirox in comparison to deferoxamine. As regards deferiprone, some serious side effects (neutropenia, agranulocytosis) were reported (Tricta et al., 2016), possibly clarifying the slight but steady decrease in its prevalence from 2013 (25.3%) to 2018 (12.4%) (Figure 1C). As for the intensity of use, the decreasing trend observed for deferoxamine from 2015 until the end of the study period is probably the consequence of the different route of administration (subcutaneous vs. oral) and dose compared to the two other medications (Figure 1D). Of note, the prevalence of use is probably slightly underestimated, as the three drugs have specific therapeutic indications for β-thalassemia major and intermedia, while the patients of our cohort were collected within the wider unspecific group of thalassemias, which also included α-thalassemia. Nonetheless, we expect a very limited underestimation as almost all patients were affected by β-thalassemia, recognized as the most frequent in the Mediterranean area (Kattamis et al., 2020).

Eltrombopag is an oral thrombopoietin receptor agonist that represents a promising second-line treatment in the field of nontransplant therapy for patients with SAA refractory to immunosuppressive therapy (McCormack, 2015). Acquired SSA is a rare disorder of unknown etiology in most cases and presenting as immune destruction of hemopoietic stem cells (Bacigalupo, 2017). Immunosuppressive agents, namely intravenous horse antithymocyte globulin and oral cyclosporine A, given alone or in combination, are currently the recommended standard therapy for both patients with no severe disease and those with SAA who are ineligible for allogeneic hematopoietic stem-cell transplantation or lack a suitable bone marrow donor (Peslak et al., 2017). This explains the high prevalence of use of cyclosporin A observed in our cohort. On the other hand, the efficacy and the mild side-effects of eltrombopag may account for the decreasing trend in cyclosporine A use observed in the years 2016–2018, along with the recent finding that eltrombopag has also proved to be an effective first-line therapy in addition to immunosuppressive treatment (McCormack, 2015; Townsley et al., 2017).

### Metabolic Disorders

In UCDs, NaPB belongs to the pharmaceutical class of ammonia-scavenging drugs, which prevent the accumulation of ammonia by providing an alternative pathway for nitrogen disposal, thus favoring the metabolic control of the disease (Peña-Quintana et al., 2017). NaPB does not cause serious adverse events such as liver injury, thus representing an improvement in UCDs therapy (Peña-Quintana et al., 2017). However, gastrointestinal disorders and metabolism and nutrition disorders are common side effects related to the unpleasant taste of the drug (Peña-Quintana et al., 2017; EMA, 2021e). Besides, amenorrhea and irregular menstruation can occur quite frequently in fertile women (EMA, 2021e). The presence of these side effects can only partially explain the low overall prevalence of use observed in our cohort (Figure 2A). Actually, for most patients, the long-term management of UCDs relies on a low-protein diet and supplementation of arginine and/or citrulline. Instead, in subjects requiring medications to increase waste nitrogen excretion, oral sodium benzoate, although not a registered drug, is a first-line medication in UCDs in many centres in Europe, including the Tuscany Metabolic Centre. In particular, sodium benzoate has less side effects, fewer safety concerns, and lower price compared to the novel tasteless orphan drug, glycerol phenylbutyrate (Häberle et al., 2019). On the other hand, NaPB is used as adjunctive medication, and to overcome the critical issues related to the bitter taste of NaPB capsules, a new taste-masked, odorless and slow-release granule formulation has been developed and approved by EMA in 2013 (Häberle et al., 2019; EMA, 2021f). Indeed, the prevalence of use slightly increased since 2014, up to 11.8% in 2018 (Figure 2A). As regards the decreasing intensity of use of this drug observed in this study from 2016 onwards, it may be attributable to the liver transplantation occurred for some patients, which provides a definitive cure and allows for a normal diet without taking nitrogen scavengers (Häberle et al., 2019) (Figure 2B). MMA is an autosomal recessive inherited rare disease of metabolism involving pathogenic variants of the genes encoding for methylmalonic-CoA mutase (MMA *mut* type) or involved in the synthesis of its cofactor adenosylcobalamin (MMA *cbl* type) (Chakrapani et al., 2018; Nashabat et al., 2019). The consequent accumulation of methylmalonic-CoA results in the competitive inhibition of N-acetylglutamate synthase and carbamoyl phosphate synthase 1 (CPS 1), an enzyme involved in the first and rate-limiting step of the urea cycle, causing hyperammonemia (Alfadhel et al., 2021). Carglumic acid restores the function of the urea cycle by activating CPS 1 and normalizes blood ammonia levels during acute decompensation episodes (Chakrapani et al., 2018; Nashabat et al., 2019). As regards the other non-orphan medications, levocarnitine is administered to compensate for secondary carnitine deficiency caused by urinary loss of carnitine-bound to organic acids in all patients affected by MMA *mut*; hydroxocobalamin is given in responsive MMA *cblC* patients to increase serum methionine levels, while betaine is indicated to increase the re-methylation of homocysteine to methionine in MMA *cblC* (Baumgartner et al., 2014; Forny et al., 2021). All these medications are safe and generally well-tolerated, but they have different therapeutic indications, hence a direct comparison on their prevalence of use would lead to incorrect evaluations. Current guidelines recommend the use of carglumic acid during decompensation episodes, which carry a high risk of mortality and neurological complications if not promptly treated (Valayannopoulos et al., 2016; Häberle et al., 2018; Nashabat et al., 2019), whereas there is still limited evidence on the long-term efficacy of carglumic acid in MMA (Alfadhel et al., 2021). In addition, the frequent poor adherence of patients to carglumic acid and its current use mainly in hospitalized patients with severe disease may further account for the low prevalence of this orphan drug use found in our cohort. Given the impossibility of collecting inpatient prescriptions, the prevalence of use of carglumic acid is therefore probably underestimated.

Nowadays, three main treatments are available to lower plasma phenylalanine (Phe) levels in PKU, which is caused by mutations in the Phe hydroxylase (PAH) gene or by the deficiency of tetrahydrobiopterin (BH4) that acts as PAH cofactor (Blau et al., 2021). These defects, if not treated, may produce intellectual disability (Blau et al., 2021), and while the low-Phe diet, along with early diagnosis, was the only treatment strategy for managing PKU until 2007, the recent development of chaperone and enzyme replacement adjunctive therapies sometimes allows for the discontinuation of the low-Phe diet (Lichter-Konecki and Vockley, 2019). Sapropterin dihydrochloride, introduced in the EU in 2008, is a synthetic form of BH4 and, as such, exerts a function of molecular chaperone promoting correct folding and stability of mutant misfolded PHA (Lichter-Konecki and Vockley, 2019). Sapropterin dihydrochloride can lead to a significant decrease in plasma Phe concentration (Levy et al., 2007; Trefz et al., 2009) and has also an acceptable safety profile, with mild side effects (EMA, 2021g). Nonetheless, it is more likely effective in reducing plasma Phe levels in patients who respond to pharmacological doses of BH4 or in those with milder forms of PKU (Lichter-Konecki and Vockley, 2019; Blau et al., 2021). While the complete suppression of PAH activity is more common, the low rate of prevalence of sapropterin dihydrochloride found in this cohort (Figure 2D) could be attributable both to the fewer BH4 responders observed in populations (van Spronsen et al., 2017) and to the good efficacy and compliance with long-term low Phe diet of these patients who have expressed no interest in changing therapy (Lichter-Konecki and Vockley, 2019).

Due to their oral administration, miglustat and eliglustat are generally better accepted than ERT to treat Gaucher disease, an inherited lysosomal storage disorder caused by deficiency or absence of the activity of acid β-glucosidase, by rebalancing glucocerebroside metabolism (Hollak, 2012; Belmatoug et al., 2017; Biegstraaten et al., 2018). Miglustat is indicated for the treatment of patients with mild-to-moderate GDI and, as it presents many adverse effects, some of which very frequent (e.g., diarrhea, weight loss, peripheral neuropathies, tremor, and cognitive disorders), preferably it has not been used since 2010 in Tuscany (EMA, 2021h). Eliglustat is a glucosylceramide analogue acting as an inhibitor of glucosylceramide synthase and, compared to miglustat, it has a high therapeutic index with limited toxicity (Cox et al., 2015), however it was not possible to evaluate its trend of use as it was only prescribed in the last year of the study period. The low prevalence of use observed in this study for both orphan drugs is probably a consequence of the widespread use in clinical practice and the elevated efficacy of ERT in improving quality of life and many outcomes of GDI, such as regression of organomegaly, reversal of anemia and thrombocytopenia, and amelioration of bone pain (Biegstraaten et al., 2018).

Fabry disease is an X-linked progressive lysosomal disorder caused by a deficiency or absence of α-galactosidase A (α-Gal A) activity, accumulation of glycosphingolipid in the plasma and other tissues and consequent life-threatening complications, especially in the heart, kidneys, and central nervous system (CNS) (Germain et al., 2019; McCafferty et al., 2019). Migalastat is an oral pharmacological chaperone able to promote enzyme folding and stability, and to restore endogeneous α-Gal A activity (McCafferty et al., 2019). It may overcome some limitations of ERT, such as frequent infusions required and associated local reactions, anti-drug antibody production, and high impact on quality of life (Parenti, 2009). Furthermore, being a small molecule, migalastat may produce enhanced cellular and tissue distribution and has the potential to cross the blood-brain barrier (Parenti, 2009). Migalastat, used to treat patients aged 12 years and above, is generally well tolerated and the most common associated-side effects are headache and nasopharyngitis (McCafferty et al., 2019; EMA, 2021i). Hence, migalastat represents a promising therapeutic avenue and, although ERT is the first treatment approved for Fabry disease and its efficacy ascertained (Miller et al., 2020), the prevalence of use of migalastat appears to be growing. The clinical outcome of this trend needs to be evaluated in the next few years.

None of the available therapies for WD, an autosomal recessive disease characterized by excessive accumulation of copper in the liver and brain, can decisively cure the disorder, however all have improved survival of patients, especially in the case of early diagnosis and therapy (Chang et al., 2013; Gupta et al., 2018). In particular, zinc acetate is an oral copper-lowering drug that blocks dietary copper absorption in the intestinal tract by increasing the production of cellular metallothionein (Aggarwal et al., 2018; Appenzeller-Herzog et al., 2019). D-penicillamine is currently the standard of care for patients with WD in most countries, being the recommended therapeutic option in symptomatic subjects both during the initial intensive phase of treatment and later as lifelong therapy (Chang et al., 2013). Zinc acetate is instead advocated as maintenance treatment in patients previously treated with other copper chelators and presenting with regression of symptoms (Roberts et al., 2008). In monotherapy or in combination with penicillamine, zinc has further demonstrated efficacy and safety in young children (Mizuochi et al., 2011; Chang et al., 2013). Although there is still insufficient evidence to claim superiority of one WD treatment and all available anti-copper therapies can be associated with neurological worsening in a subset of WD patients (Samanci et al., 2021), zinc has fewer side effects and lower treatment discontinuation rate than penicillamine therapy (Członkowska et al., 2014; Litwin et al., 2015; Appenzeller-Herzog et al., 2019).

As discussed for WD, also for LHON, a maternally inherited mitochondrial disorder characterized by progressive bilateral vision loss, there are no definitely curative or high-effective therapies (Karaarslan, 2019). Idebenone is an antioxidant agent related to coenzyme Q10 (ubiquinone), which likely restores cellular energy generation, reduces oxidative stress, and prevents apoptosis of retinal ganglion cells (Lyseng-Williamson, 2016; Karaarslan, 2019). The efficacy, safety and good tolerability of idebenone were documented, with only mild to moderate associated-side effects commonly reported (Klopstock et al., 2011; 2013; Lyseng-Williamson, 2016). Clinical practice has also indicated a beneficial effect of idebenone on improving visual function (Cheng et al., 2014), although once the disease has become chronic, the efficacy of idebenone is yet to be ascertained (Pemp et al., 2019). This could explain the utilization profile (Figure 3C) that depends both on the phase of disease in which the diagnosis is made and on the poor efficacy of idebenone in end-stage LHON. Furthermore, the decreasing trend in the prevalence of use may be due to the progressively lower number of new subjects treated per year once all previous diagnosed patients have been treated.

### Immune System Disorders

Therapeutic options for patients with HAE embrace on-demand treatment to revert attacks and long-term prophylaxis treatment to prevent recurrences (Perego et al., 2019). In most cases, HAE is characterized by reduced production and/or functional activity of the C1-esterase inhibitor (C1-INH), which results in the activation of the plasma cascade system and the generation of bradykinin (Farkas et al., 2020). Icatibant acts as a selective and specific antagonist of the bradykinin receptor, inhibiting bradykinin-induced vasodilation in humans (Perego et al., 2019), and it is considered an early treatment associated with a shorter total attack duration, a shorter time to onset of symptom relief and the prevention of severe outcomes (Maurer et al., 2013). Efficacy and safety of icatibant were demonstrated in patients with acute HAE attacks (Malbrán et al., 2014; Farkas et al., 2017). Icatibant-treated subjects can experience gastrointestinal symptoms and injection-site reactions, although no serious adverse events have been reported (Lumry et al., 2011; Farkas et al., 2017). Nevertheless, C1-INH replacement by the use of human C1-INH concentrate, inhibiting pathways leading to bradykinin production, is an effective treatment and is recommended as first-line therapy for acute edema attacks in patients with HAE (Craig et al., 2013; Maurer et al., 2013; Martinez-Saguer et al., 2014). Efficacy, safety and tolerability, opportunity of weight-adjusted doses and indications in pre-operative prophylaxis of pdC1-INH (Maurer et al., 2018), along with the elevated costs of icatibant, might provide possible explanations for the generally higher prevalence of the non-orphan drug in our patient population (Figure 4A). On the other hand, the low Pr/Us values observed (Figure 4B) confirmed that both drugs are employed in the management of acute disease (Craig et al., 2013).

### Respiratory Diseases

IPF is a chronic, progressive, fibrosing interstitial pneumonia (Maher and Strek, 2019). Even though pirfenidone, an oral antifibrotic drug, is not capable of halting disease progression, it may reduce decline in lung function by inhibiting proliferation, myofibroblast differentiation and fibrogenic activity of primary human lung fibroblasts (Conte et al., 2014; King et al., 2014), resulting in a significant improvement in terms of life expectancy (Zurkova et al., 2019). Pirfenidone is generally considered safe with an acceptable side-effect profile, except for gastrointestinal symptoms, rash and photosensitivity, and elevation of liver enzymes (Maher and Strek, 2019). In the past, azathioprine, methylprednisolone and prednisone, and N-acetylcysteine had been administered alone or in combination to slow functional deterioration in patients with IPF (Demedts et al., 2005; Peikert et al., 2008). Nonetheless, in 2012 the triple therapy with prednisone, azathioprine and N-acetylcysteine was demonstrated to significantly increase all-cause mortality, all-cause hospitalizations and treatment-related severe adverse events (Behr, 2012). Consistent with its efficacy and safety profile, pirfenidone showed an increasing prevalence of use since 2013, apparently in substitution of glucocorticoids, whereas the decreasing trend of azathioprine appeared to be independent of the orphan drug’s introduction. Accordingly, also the prescription rate of N-acetylcysteine showed a progressive decline (Figure 5A). These findings are probably related to the evidence of detrimental or not significant beneficial effects in lung function decline and adverse events rate determined by glucocorticoids, azathioprine and N-acetylcysteine, opposed to the efficacy of pirfenidone in reducing disease progression rate, which led to the first approval for a pharmacological treatment in IPF.

The orphan drugs bosentan, ambrisentan and macitentan, authorized for marketing in Italy in 2001, 2008 and 2013, respectively, are endothelin receptor antagonists (ERAs), exhibiting different affinity degree for endothelin receptors and recommended in the EU for the long-term treatment of adults with pulmonary arterial hypertension (PAH) in the World Health Organization-Functional Classes II-III (WHO FC II, FC III) (Keating, 2016; Mayeux et al., 2021). Endothelin-1 is a key mediator of PAH, causing proliferation and vasoconstriction in pulmonary vascular smooth muscle cells, and proliferation and vasodilation in pulmonary endothelial cells (Sitbon and Morrell, 2012). Macitentan, derived from the structure of bosentan, is generally well tolerated (Keating, 2016; Belge and Delcroix, 2019) and was reported to significantly reduce morbidity and mortality (Pulido et al., 2013; Kim et al., 2016). The most frequent adverse events associated to macitentan included anemia, nasopharyngitis, bronchitis and headache (Belge and Delcroix, 2019), whereas adverse effects on liver function were observed for bosentan (Keating, 2016; Kuang et al., 2018). Compared to other ERAs, macitentan has been shown ten times more potent than bosentan in lowering blood pressure (Iglarz et al., 2008). Overall, these reasons could explain the steadily decreasing trend of bosentan, and the increase in the prevalence of macitentan since 2016, which in turn could also partly account for the slight reduction in ambrisentan use (Figure 5C). In PAH, nitric oxide (NO) production is chronically impaired, resulting in increased vasoconstriction and proliferation within vascular smooth muscle cells (Mayeux et al., 2021). Sildenafil is an inhibitor of phosphodiesterase type-5 (PDE5) and, as such, stimulates NO-dependent pathway (Galiè et al., 2015). It is typically the first-line therapy prescribed for treating patients with PAH; however, despite improved multiple clinical outcomes in patients with PAH, sildenafil appears to have no significant effects on mortality and serious adverse events (Wang et al., 2014; Mayeux et al., 2021). Accordingly, sildenafil showed a nearly steady trend under the period of investigation (Figure 5C). Riociguat, which enhances NO pathway through the direct stimulation of soluble guanylate cyclase (sGC), constitutes a valuable novel therapy for PAH, although patients may experience episodes of hypotension (Hambly and Granton, 2015; Keating, 2016). Iloprost, an inhaled orphan drug, belongs to prostacyclin analogs and exerts an anti-aggregatory, antiproliferative and vasodilative action by increasing levels of cyclic guanosine monophosphate (Kuang et al., 2019). Iloprost is an effective, safe, and well-tolerated agent for PAH in the first 3 months after diagnosis but, when used in monotherapy for a prolonged period, an unsatisfactory effect on pulmonary hemodynamics and event-free survival rate was observed (Kuang et al., 2019). The observed trend patterns are a consequence of the therapeutic strategy adopted in PAH, in addition to other factors such as date of marketing authorization, safety profile and possible interactions with other drugs, route of administration, comorbidities, patient preferences, and cost (Barberà et al., 2018). In particular, ERAs, PDE5 inhibitors and sGC stimulators in monotherapy are indicated for patients with WHO FC II and III, while prostacyclin analogs, such as iloprost, seem the most effective for the treatment of subjects with WHO FC III (Barberà et al., 2018).

### Peripheral and Central Nervous System Disorders

Rufinamide is an oral third-generation antiepileptic drug (AED), structurally unrelated to other AEDs, which acts primarily by prolonging the inactivation phase of voltage-gated sodium channels (Striano et al., 2018; Verrotti et al., 2018). Rufinamide is effective as a long-term adjunctive therapy in reducing frequency and severity of seizures associated with LGS, a severe epileptic encephalopathy (McMurray and Striano, 2016; Ohtsuka et al., 2016; Jaraba et al., 2017; Striano et al., 2018). This drug is generally well-tolerated, with mild or moderate symptoms such as somnolence, decreased appetite, vomiting, weight loss (McMurray and Striano, 2016; Ohtsuka et al., 2016). Topiramate is a second-generation AED whose antiepileptic properties depend on various biological mechanisms (e.g., blockade of voltage-dependent sodium channels, potentiation of γ-aminobutyric acid-mediated transmission, antagonism non N-methyl-D-aspartate glutamate receptor) and, like rufinamide, it is used as monotherapy or as adjunctive therapy for reducing the number of drop attacks and major motor seizures and offers a favorable side-effect profile (Mula et al., 2006; Verrotti et al., 2018). Though rufinamide exhibited a higher prevalence of use if compared to topiramate from 2012 onwards (Figure 6A), there are currently no evidence-based guidelines on the most appropriate AED therapy for patients with LGS (Rosati et al., 2018). Therefore, antiepileptic therapy should be as adapted as possible to each patient based on his clinical history, comorbidities, and type of seizure (Rosati et al., 2015).

### Congenital Anomalies, Chromosomal Aberrations and Genetic Syndromes

TSC results from mutations in TSC1 and TSC2 genes, which encode, respectively for the tumors suppressors hamartin and tuberin, leading to the constitutive activation of the mechanistic target of rapamycin (mTOR) signaling pathway and uncontrolled growth and proliferation (Krymskaya, 2003; Luo et al., 2021). Both everolimus and sirolimus are mTOR inhibitors employed as the primary modality for the management of TSC-associated renal angiomyolipomas (AMLs) and subependymal ependymal giant cell astrocytomas (SEGAs) in the CNS (Ebrahimi-Fakhari and Franz, 2020; Luo et al., 2021). Everolimus has also indications for additional treatment of seizures related to TSC in patients older than 2 years of age that have not responded to other treatments (EMA, 2021j). Although EMA-approval of sirolimus in TSC is limited for pulmonary lymphangioleiomyomatosis (EMA, 2021k), the two medications are used interchangeably in clinical practice due to their ability to improve the clinical status of TSC patients and to significantly reduce the tumor volume in SEGA and AML (Ebrahimi-Fakhari and Franz, 2020; Luo et al., 2021). Furthermore, they have both significant adverse effects with no statistical differences, most of which tend to be mild (Li et al., 2019; EMA, 2021j). Overall, this may explain the comparable prevalence of use found in our cohort for the two medications (Figure 6C).

### Strength and Limitations

This study used a population-based registry that collects all cases diagnosed with one of the rare diseases defined by the Italian Law, in a residing population of approximately 3,7 million inhabitants. The analysis covers a 10-year period, including patients with a rare disease surveilled by the registry from 2000 onwards, which is long enough to provide the current profile of orphan drug use in a large cohort. The use of a multi-database approach, integrating data collected by a population-based registry with prescription data obtained from health administrative databases, allows to take advantage of the ability of a population-based registry to collect almost all patients diagnosed in a defined geographical area and, at the same time, exploit information about prescriptions and therapies routinely collected at the local level.

This study has some limitations. First, since the Tuscany Registry of Rare Diseases is active since 2005, diagnoses collected retrospectively for the previous years (2000–2004) might be underestimated. Second, only the diseases monitored by the Tuscany Registry of Rare Disease were included this study, namely those reported in the list of the Italian Law and for which an exemption for co-payment is provided. Third, the prevalence and intensity of use of orphan drug for certain rare diseases (i.e., β-thalassemia, Gaucher disease type I) could be under-reported due to inaccurate correspondence between the specific therapeutic indication for the orphan drug and the definitions of rare disease surveilled by the Italian Law (e.g., deferasirox has therapeutic indication for β-thalassemia but the Tuscany Registry of Rare Diseases collects both cases of α- and β-thalassemia). Fourth, with respect to different coagulation factors IX used for the treatment of hemophilia B, they may have different dosages, which could lead to a distortion of the general calculation of the intensity of use. Fifth, a slight underestimation of the prevalence and intensity of use of orphan drug cannot be excluded for rare diseases sometimes characterized by long hospitalizations, as in the case of methylmalonic acidemia, since the available pharmaceutical databases do not register inpatient prescriptions. Finally, given the exclusion from the analysis of drugs mainly used in inpatient setting or not traceable from our pharmaceutical databases for other reasons (e.g., specialistic prescriptions), for certain diseases (acquired aplastic anemia, urea cycle disorders, Gaucher disease type I, Fabry disease, Wilson disease), the percentage of treated patients might result underestimated.

## Conclusion

To the best of our knowledge, this is the first study that investigated the profile of use of orphan drugs in a defined geographical area. It explored the prevalence and intensity of use of 21 orphan drugs indicated for a total of 15 rare diseases, over a 10-year period.

Overall, there is at least one orphan medicinal product for each rare disease included in the study, two orphan drugs for urea cycle disorders, phenylketonuria, Gaucher disease type I, Wilson disease and idiopathic pulmonary fibrosis, and six medications with orphan designation for pulmonary arterial hypertension. For hemophilia B and Leber’s hereditary optic neuropathy, there are currently no other medications used in clinical practice in addition to orphan drugs.

In this study, the use of orphan drugs was higher than the other available pharmacological treatments in Lennox Gastaut syndrome and idiopathic pulmonary fibrosis, while, albeit with different profiles of use, orphan medications appeared the elective therapeutic option in pulmonary arterial hypertension. In contrast, a low prevalence of use of orphan medicinal products was observed in acquired aplastic anemia, most metabolic diseases, and tuberous sclerosis complex, probably as a consequence of greater adherence of patients to conventional therapies and, in some cases, of the short period under examination from the approval of the orphan drugs (i.e., eliglustat in Gaucher Disease type I and migalastat in Fabry disease). On the other hand, despite a very low prevalence of use in some rare diseases (e.g., urea cycle disorders), orphan drugs ensure milder side effects than conventional standard of care, elevated safety profile, besides a generally friendly route of administration. Other factors such as marketing strategies and elevated costs may also influence the utilization profile of orphan drugs.

This study provides an overview of the orphan drug use profile in an Italian region over a 10-year period using population-based and health administrative data. This multi-database system also represents a useful epidemiological surveillance tool to monitor over time potential changes in the prevalence of orphan drugs already authorized for marketing, as well as that of other orphan medicinal products to be approved in the coming years. Furthermore, these data may provide a useful insight into healthcare decision-making about efficacy of medications for orphan diseases in the clinical setting and, at the same time, promote a more in-depth analysis concerning the cost-effectiveness of orphan drugs.

## Data Availability

The data supporting the findings of this study are available from Regione Toscana but restrictions apply to the availability of these data, which were used under license for the current study, and therefore are not publicly available. Data are however available from the authors upon reasonable request and with permission of Regione Toscana. Requests to access the datasets should be directed to Regione Toscana, https://www.regione.toscana.it/.

## Glossary

AED: Antiepileptic drug
AML: Angiomyolipoma
ATC: Anatomical therapeutic chemical
α-Gal A: α-galactosidase A
BH4: Tetrahydrobiopterin
C1-INH: C1-esterase inhibitor
CNS: Central nervous system
CPS 1: Carbamoyl phosphate synthase 1
DDD: Defined Daily Dose
ERAs: Endothelin receptor antagonists
ERT: Enzyme replacement therapy
EMA: European Medicines Agency
FD: Fabry disease
FIX: Coagulation factor IX
GDI: Gaucher disease type I
HAE: Hereditary angioedema
IPF: Idiopathic pulmonary fibrosis
LGS: Lennox-Gastaut syndrome
LHON: Leber’s hereditary optic neuropathy
MMA: Methylmalonic acidemia
mTOR: mechanistic target of rapamycin
NaPB: Sodium phenylbutyrate
NO: Nitric oxide
PAH gene: gene encoding for Phenylalanine hydroxylase
PAH: Pulmonary arterial hypertension
PDE5: Phosphodiesterase type-5
PKU: Phenylketonuria
pdC1-INH: Plasma-derived C1-esterase inhibitor
Pr/Us: Prescriptions per user
SAA: Severe aplastic anemia
SEGA: Subependymal ependymal giant cell astrocytoma
sGC: Soluble guanylate cyclase
SRT: Substrate reduction therapy
TSC: Tuberous sclerosis complex
UCD: Urea cycle disorder
WD: Wilson disease
WHO-FC: World Health Organization-Functional class.
